# Potential of Industrial Hemp for Phytoremediation of Heavy Metals

**DOI:** 10.3390/plants11050595

**Published:** 2022-02-23

**Authors:** Dante F. Placido, Charles C. Lee

**Affiliations:** USDA-ARS-Western Regional Research Center, Bioproducts Research Unit, 800 Buchanan St., Albany, CA 94710, USA; dante.placido@gmail.com

**Keywords:** *Cannabis sativa*, hemp, phytoremediation, polluted soil, bioengineering

## Abstract

The accumulation of anthropogenic heavy metals in soil is a major form of pollution. Such potentially toxic elements are nonbiodegradable and persist for many years as threats to human and environmental health. Traditional forms of remediation are costly and potentially damaging to the land. An alternative strategy is phytoremediation, where plants are used to capture metals from the environment. Industrial hemp (*Cannabis sativa*) is a promising candidate for phytoremediation. Hemp has deep roots and is tolerant to the accumulation of different metals. In addition, the crop biomass has many potential commercial uses after harvesting is completed. Furthermore, the recent availability of an annotated genome sequence provides a powerful tool for the bioengineering of *C. sativa* for better phytoremediation.

## 1. Introduction

Heavy metals have a wide variety of industrial applications, but their intensive production and use come at a price: adverse effects on human health and on the vast lands producing the crops that feed the world [1,2,3]. The relevance and urgency of the aforementioned problem increase as modern industry increasingly relies on advanced technologies for sustainability, and many of these technologies require potentially toxic metals. For instance, cadmium (Cd), lead (Pb), and nickel (Ni) are used in renewable technologies such as battery cells, solar cells, and electric-car batteries [2]. Furthermore, as the human population continues to grow, so does our dire need for more fertile land to grow crops. Metals from the mining and smelting of metalliferous ores and downwash from power lines, municipal wastes, fertilizers, pesticides, and sewage have left much land too toxic to cultivate crops [1,2]. Metals do not degrade; they can only be altered from one oxidation state or organic complex to another [4]. Typical strategies to remediate polluted soils are expensive and environmentally damaging. Examples include the excavation and burial of polluted soil at hazardous waste sites, the chemical processing of soil to immobilize metals, and using acid solutions to desorb and leach metals from soil taken from a waste site and returning the clean soil residue to the site.

Phytoremediation is a feasible, economical, and sustainable alternative for cleaning polluted soil. Phytoremediation is the use of green plants to remove metal pollutants from soil or render them harmless [5,6,7,8]. Many plants are known to accumulate metal pollutants, and the ability to accumulate metal varies significantly between species and between cultivars within a species. Most metal-accumulating plants are small shrubs with shallow roots requiring specific growing conditions [9,10,11,12,13]. To be effective at soil remediation, more than just the top layer of soil needs to be cleaned of pollutants. Hemp’s ability to extract metals from soil with its deep roots, combined with its commercial prospects, make it an ideal candidate as a profit-yielding crop when used for phytoremediation purposes.

Industrial hemp (*Cannabis sativa*) is a naturally dioecious, flowering plant, where the male and female reproductive organs are located on separate plants [14]. Monoecious cultivars also exist, where the male and female organs are carried on the same plant. Hemp grows best at temperatures ranging from 16 to 27 °C [15]. Hemp is a short-day plant, requiring less than 12 to 14 h of sunlight to flower [16]. The plants are tall (~6 m) and have deep roots (45–90 cm) [15]. The flowers take 10 to 16 weeks to mature, and fiber production is at its highest at the full-flowering stage [17]. At full flowering, fiber maturation at higher internodes is more advanced and fiber is more homogenous.

Hemp has very good prospects as a phytoremediator: not only can it be grown on contaminated sites, but it also produces marketable products used for bioenergy production, timber fiber, pulp, and fodder [18,19,20,21]. Hemp is used to clean up metals, pesticides, solvents, explosives, crude oil, polyaromatic hydrocarbons, and toxins. Hemp harvested from remediation sites can be safely distilled into ethanol for use as a biofuel [19,20,21]. The conversion of biomass to biofuels and chemicals has traditionally been from food crops such as corn, wheat, sugar beets, and sugar cane, but lignocellulosic biomass has generated much interest as a promising, renewable source of bioethanol because of its wide availability and low cost of feedstocks [19]. A comparative cost analysis indicated that hemp is a profitable commodity crop for producing both biofuels and value-added products [22].

The fact that hemp accumulates potentially toxic metals in all plant parts limits its use as a raw material in clothing and the food chain. Several processes can remove metals from leaves and stems that have high concentrations of cadmium, lead, or nickel. One method uses thermochemical pretreatment prior to enzymatic hydrolysis for the bioconversion of hemp biomass into succinic acid [23]. Succinic acid is used as a precursor for biodegradable polymers, food, fine chemicals, green solvents, and pharmaceuticals [24]. Hemp-hurd biomass along with *Ralstonia eutropha* are utilized for the production of poly-3-hydroxybutyrate [25]. The high quality of hemp fibers and hurds is not affected by metal contamination, allowing them to be used in products such as composite materials. Following the harvest of pollutant-enriched plants, contaminated material is either composted, disposed of as hazardous material, or (more economically interesting) used for metal recovery [26]. Furthermore, hemp fibers can be used in sustainable polymer matrix composites because they are renewable [27,28,29]. The December 1941 edition of Popular Mechanics Magazine reported that hemp fibers were used in resin matrix composites for the bodywork of Henry Ford’s car, which was claimed to have an “impact strength 10 times greater than steel”.

## 2. Phytoremediation

### 2.1. Phytoremediation Process

Potentially toxic metals first interact with plants at the roots, where they are taken up by mass flow and diffusion. These metal pollutants are made bioavailable for plants through root secretion of metal-chelating molecules into the surrounding rhizosphere [30], metal reductase in the plasma membrane, and proton extrusion from roots [26]. Several mechanisms of phytoremediation exist [2,9,31,32]. In phytoextraction, soil contaminants are taken up through the roots and accumulate in the shoots [33,34]. In general, higher concentrations of metal in the growth environment result in higher accumulations in plant tissue [35,36,37,38,39,40]. Then, the contaminated shoot tissues are processed using a variety of disposal methods, such as heat and extraction treatments [41]. For instance, the tissues may be harvested and incinerated as hazardous waste, with the ash being discarded in landfills [42,43], or utilized for the re-extraction of trace elements [44,45]. The harvested biomass can alternatively be used as feedstock for bioenergy production or pyrolyzed to form biochar [46,47,48,49]. Phytostabilization is a process in which metallic contaminants are immobilized through root adsorption and metal precipitation and stabilized through complex formation or reduction [50]. The immobilization and stabilization of metals to a nontoxic form within the plant prevents interference with cellular metabolism [51]. Phytovolatilization converts potentially toxic metals to more-volatile forms that are removed to the atmosphere through transpiration [44].

### 2.2. Hemp as a Phytoremediator

Hemp grows quickly and has deep, wide roots [15]. It can adapt to different soil conditions and grows in a variety of climates [36]. Many studies showed that hemp has a high tolerance to metals [52]. Industrial hemp can often take up metals and store them in different parts of the plant, with no detriment to the plant itself (Table 1) [36,53,54]. When employed for phytoremediation purposes, toxins can accumulate in the roots, leaves, and stalks [35]. Therefore, the leaves are not harvested for food or used for personal care; however, the stalks can be utilized for building materials, paper, cloth, and biofuel [55]. Since 1998, hemp has been successfully used to remove soil contaminants from agricultural lands that were heavily contaminated by the 1986 Chernobyl nuclear disaster [56]. In 2008, in an Italian farming region contaminated by a nearby steel plant, hemp was grown to leach pollutants, such as dioxin, from the soil [57]. Dioxins are considered toxic as they cause cancer, affect reproduction and development, damage the immune system, and interfere with hormones. Once remediation is complete, plant material containing dioxins can be used to produce energy. Beyond cleaning soil, research is being conducted on using hemp fibers to create absorption material capable of filtering out metals from contaminated water [58].

### 2.3. Chromium Phytoremediation

In hemp, chromium (Cr) is absorbed passively with other essential metals [61]. Chromium accumulates significantly in the root system (for both seed and fiber varieties of hemp) and less so in the stems, leaves, and seeds [36,38,59,60]. As observed in other plants, hemp’s ability to immobilize Cr in the vacuoles of root cells may explain its high accumulation in the roots of the plant [61,62]. A higher amount of Cr in the roots of hemp from contaminated sites leads to significantly higher proline accumulation and an increase in the phenolics content. Both proline and phenolics accumulation prevent oxidative injury in plants. At the same time, when Cr accumulates in root systems, chlorophyll, carotenoids, and dry biomass decrease significantly [60].

### 2.4. Zinc Phytoremediation

Hemp can tolerate high concentrations of zinc (Zn), and most of the Zn absorbed by hemp was retained in the roots [39]. Zn shoot restriction means minimal damage to photosynthetic activity and healthy plant growth. Low concentrations of Zn are also found in seeds, making them suitable for alimentary use [52,63]. In contrast, Malik et al. found that Zn accumulates at higher levels in shoots than in roots [59]. Angelova et al. reported Zn concentrations in the following order: flower > seeds > roots > stems > leaves > fiber [35].

### 2.5. Copper Phytoremediation

In hemp, copper (Cu) accumulates in the leaves but not in the fibers [64]. Glutathione-disulfide reductase (GSR) and phospholipase D-α (PLDα) are major antioxidant enzymes that protect plant cells against oxidative damage caused by reactive oxygen species (ROS) produced under metal-stress conditions. The expressions of GSR and PLDα were found to be induced in hemp that have accumulated high concentrations of Cu [52]. Other studies have reported an increase in aldo-keto reductase, an NAD(P)H-dependent enzyme, in hemp grown under Cu stress [65,66]. This reductase is involved in the detoxification process by improving the scavenging capacity of the cell [67]. Authors proposed that this protein reduces ions, making them available for interaction with other proteins, such as phytochelatins, that can transport them to the vacuole [65]. Although photosynthesis was not affected in that study, Cu treatment resulted in a significant reduction in the aerial parts of the plant as well as the root-system architecture. Angelova et al. reported Cu concentrations in the following order: flower > seeds > roots > stems > leaves > fiber [35].

### 2.6. Selenium Phytoremediation

Hemp can grow in selenium (Se)-laden soil and accumulates selenomethionine and methylselenocysteine in seed embryos [40]. This means that the seeds can be used as a Se supplement for humans as well as livestock. Se is also present in other above-ground parts. Se found in the flower (where CBD and terpenes are produced and extracted) and stems (where fiber is produced) has no negative effect on the yield of such metabolites or on fiber quality. Se in the leaves can be applied as fertilizer for crops growing in low-Se soil.

### 2.7. Cadmium, Nickel, and Lead Phytoremediation

Hemp can take up high concentrations of cadmium, nickel, and lead from metal-contaminated environments, such as soil from abandoned mines [37]. For each of these metals, their concentration was highest in the leaves. In another study with the same metal contamination, the quality of fibers and hurds was not affected, which allowed them to be used in products like composite materials [53]. Growing in soil with high concentrations of Cd has no negative effect on hemp germination, but as the plants mature, they accumulate Cd in aerial parts, negatively influencing photosynthesis and inhibiting plant growth [35]. Surprisingly, Cd accumulation was highest in the roots, but root growth was not inhibited. Ahmad et al. also demonstrated that Pb accumulated mainly in the leaves [52], although Angelova et al. reported Pb concentrations in the following order: flower > roots > stems > leaves > seeds > fiber [35].

## 3. Bioengineering

### 3.1. Genome

The *C. sativa* genome sequence is now available. This plant has a diploid genome (2n = 20) composed of nine autosomes and a pair of sex chromosomes (X and Y). The updated genome assembly reports that the wild-type variety, *C. sativa*, genome is approximately 808 Mb, with over 38,000 protein-coding genes functionally annotated [68]. The genome sequence lays the foundation for the elucidation of molecular pathways involved in metal tolerance and will, therefore, prove invaluable for engineering hemp plants that grow better on land contaminated by potentially toxic metals. For example, proteomic studies combining ionomics and genomics approaches revealed mechanisms used by hemp to tolerate Cu [65,66]. This wealth of genomic data is vital in directing the genetic engineering of hemp plants.

### 3.2. Transgenic Plants

Bioengineering transgenic plants to improve their ability to remediate metal pollution in the field is a promising strategy [69,70]. The creation of transgenic plants involves the transfer and insertion of a desirable gene from a foreign source into a plant of interest [71]. After DNA recombination occurs, the foreign gene becomes heritable and confers improved traits on the plant. For example, metal stress leads to the production of ROS, resulting in oxidative stress; therefore, one strategy to enhance metal tolerance is to overexpress genes involved in antioxidant machinery [72]. In order to improve the capacity to accumulate metals, genes involved in uptake, translocation, and sequestration can be overexpressed [73,74]. Only a few metals, such as Zn and Cd, exist as soluble components in soil and are readily available for absorption by plants. To target metals with low bioavailability, plants can be engineered to improve the innate ability of roots to secrete compounds that mobilize ions by lowering soil pH or creating complexes with metals. Another strategy is to increase the translocation of metals by overexpressing chelators that facilitate movement from the roots to the shoots and mediate intracellular sequestration into vacuoles [75].

### 3.3. Mutagenesis

Transgenic approaches raise concerns, such as accidental gene flow from foreign species to close relatives, which is why such plants are scrutinized by regulatory agencies and may be spurned by consumers [76,77]. An alternative approach is the application of mutagenesis, which induces random mutations into the plant genome [78,79]. Two widely known mutagens that have been used for crop improvement are ethyl methanesulfonate (EMS) and fast neutron (FN) bombardment [80,81]. The most-used plant material is seed. EMS is less destructive, causing point mutations, base insertions, and small deletions in DNA, whereas FN bombardment causes translocations, chromosome loss, and large deletions [78,82,83,84,85]. One disadvantage of the mutagenesis strategy is that deleterious modifications are possible because the method does not use a targeted approach [86].

### 3.4. Genome Editing

The advent of genome editing technologies such as transcription-activator-like effector nucleases, zinc-finger nuclease, and CRISPR/Cas-mediated gene mutation via nonhomologous end joining are revolutionary and precise tools for genetically engineering desired mutations in plants [2,87,88,89,90]. These methods modify the genome through insertions and deletions. A variant of the CRISPR/Cas assembly, the base-editing system, can generate single-base changes or single-nucleotide polymorphisms [91,92]. These genome editing technologies allow for a level of precision that is not possible with a mutagenesis strategy that results in numerous random mutations. Critically, the cisgenic approach embodied in these genome-editing methods makes the products subject to less regulation and more amenable to public acceptance as it does not introduce exogenous genes [88,93].

## 4. Conclusions

Industrial hemp is a very promising candidate for the phytoremediation of metal-contaminated soils. Multiple studies have demonstrated metal uptake by hemp under a variety of settings. These studies used very different conditions (growth conditions, tissues sampled, metals analyzed, and phenotypes characterized), making it difficult to develop a clear understanding of how efficiently different hemp cultivars can take up metals. There is an urgent need for more systematic studies to directly compare the concentration and tissue localization of metals in different cultivars under the same experimental conditions. Hemp culture collections are invaluable toward this effort. For instance, the U.S. Department of Agriculture has created a hemp germplasm collection that will be capable of distributing hundreds of different, well-characterized cultivars to the public within a few years. Finally, targeted genetic modifications of hemp may dramatically boost the phytoremediation effectiveness of this crop. At this time, reliable transformation and regeneration systems are still undergoing development in hemp [94,95,96,97]; however, improved cultivars will become available as the technology matures.

## Figures and Tables

**Table 1:** Heavy-metal concentrations in hemp. Listed tissues represent those with the highest concentration of metal as reported from each of the studies.

Metal	Tissue	Concentration (mg kg^−1^)	Reference
Cr	root	6.2–100	[36,38,59,60]
Zn	flower	78.6	[35]
	root	5029.8	[39]
	shoot	43.9	[59]
Cu	flower	10.2	[35]
	root	1530	[52]
	shoot	29	[59]
Se	shoot	1300	[40]
Cd	flower	1.22	[35]
	leaf	0.38–23.2	[37,52,53]
	root	1362	[36]
Ni	leaf	1.5–123	[37,52,53]
	root	13.6–321.8	[36,59]

## Data Availability

Not applicable.
